# Patient-specific automated cerebrospinal fluid pressure control to augment spinal wound closure: a case series using the LiquoGuard®

**DOI:** 10.1080/02688697.2023.2290101

**Published:** 2024-01-04

**Authors:** Danyal Z. Khan, Kanza Tariq, Keng Siang Lee, Edward W Dyson, Vittorio Russo, Laurence D Watkins, Antonino Russo

**Affiliations:** a Department of Neurosurgery, National Hospital for Neurology and Neurosurgery, London, UK; b Wellcome/EPSRC Centre for Interventional and Surgical Sciences, University College London, London, UK; c Department of Brain Repair & Rehabilitation, UCL Queen Square Institute of Neurology, London, UK; d Bristol Medical School, Faculty of Health Sciences, University of Bristol, Bristol, UK

**Keywords:** Cerebrospinal fluid production rate, CSF leak, spinal closure, surgical innovation

## Abstract

**Objective:**

Spinal cerebrospinal fluid (CSF) leaks are common, and their management is heterogeneous. For high-flow leaks, numerous studies advocate for primary dural repair and CSF diversion. The LiquoGuard7® allows automated and precise pressure and volume control, and calculation of patient-specific CSF production rate (prCSF), which is hypothesized to be increased in the context of durotomies and CSF leaks.

**Methods:**

This single-centre illustrative case series included patients undergoing complex spinal surgery where: 1) a high flow intra-operative and/or post-operative CSF leak was expected and 2) lumbar CSF drainage was performed using a LiquoGuard7®. CSF diversion was tailored to prCSF for each patient, combined with layered spinal wound closure.

**Results:**

Three patients were included, with a variety of pathologies: T7/T8 disc prolapse, T8-T9 meningioma, and T4-T5 metastatic spinal cord compression. The first two patients underwent CSF diversion to prevent post-op CSF leak, whilst the third required this in response to post-op CSF leak. CSF hyperproduction was evident in all cases (mean >/=140ml/hr). With patient-specific CSF diversion regimes, no cases required further intervention for CSF fistulae repair (including for pleural CSF effusion), wound breakdown or infection.

**Conclusions:**

Patient-specific cerebrospinal fluid drainage may be a useful tool in the management of high-flow intra-operative and post-operative CSF leaks during complex spinal surgery. These systems may reduce post-operative CSF leakage from the wound or into adjacent body cavities. Further larger studies are needed to evaluate the comparative benefits and cost-effectiveness of this approach.

## 1. Background

Post-operative cerebrospinal fluid (CSF) leaks are a common complication after spinal neurosurgery, and may have potentially serious consequences including meningitis, prolonged hospital admission or re-admission.^1–9^ Depending on the type of operation, rates can vary from 1 to 20%.^1^^,^^2^^,^^4^^,^^8–10^ Additional risk factors likely include patient characteristics (e.g. obesity), pathology factors (e.g. tumour infiltration), operative factors (e.g. revision surgery) and surgeon-related factors (e.g. experience).^3^^,^^9–14^

The management of intra-operative and post-operative CSF leaks is considerably heterogeneous.^9^ This is due in part to the heterogeneous nature of fistulae, their variable natural history, and the paucity of high-level evidence to guide spinal surgeons.^9^^,^^15^ For complex spinal wounds, with dural defects and intraoperative CSF leaks, many authors advocate for primary dural repair (with or without artificial dural substitutes) and CSF diversion (most commonly lumbar drain placement).^1–3^^,^^9^^,^^10^^,^^15^^,^^16^ Independently, primary dural repair is associated with a failure rate of up to 10%.^2^ However, CSF diversion is cited to be particularly effective in this context, decreasing the CSF pressure differential across dural defects, and facilitating the healing of the dural breach.^1^^,^^17^ This CSF pressure varies according to the level of spinal surgery, and the position of the patient.^1^^,^^2^^,^^17^ Furthermore, there is evidence that CSF production rates are higher than traditionally thought and vary considerably. This has been demonstrated in the context of congenital disorders (e.g. Chiari malformation), intracranial pressure disorders (e.g. idiopathic intracranial hypertension) and in healthy individuals.^11^^,^^18–21^ CSF hyperproduction has also been demonstrated in the context of dural breaches, in theory, strengthening the rationale for CSF diversion.^22^^,^^23^

However, CSF diversion, most commonly via lumbar drainage, has numerous risks - including infection, under/overdrainage and restricted patient movement (need for clamping before mobilization).^24^ The LiquoGuard7® system is a smart automated CSF drainage pump which mitigates some of these risks. It offers numerous benefits over conventional drains: allowing continuous drainage (rather than intermittent); precise titration of pressure and volume thresholds; granular recording of pressures and drainage; alarm systems for system malfunctions or deranged CSF pressures; and allows patients to mobilize provided the level sensor position remains fixed on their body.^24^ It also allows the calculation of the CSF production rate. These benefits have been highlighted in the context of hydrocephalus management.^25^^,^^26^ However, the use and potential benefits of this system in the context of complex spinal wound repair are less well explored.

Thus, we sought to describe our experience with automated cerebrospinal fluid pressure and volume control, tailored to patient-specific CSF production rates, using the LiquoGuard7® system in spinal neurosurgery via an illustrative case series.

## 2. Methods

This manuscript was generated using the Preferred Reporting Of CasESeries in Surgery (PROCESS) guideline.^27^

### 2.1. Study design

A single-centre consecutive case series design was adopted and included patients undergoing complex spinal surgery, operated on between 02/2021 − 06/2022, where: 1) a high flow intra-operative and/or post-operative CSF leak was expected and 2) concurrent peri-operative CSF diversion was performed via lumbar drain attached to LiquoGuard7®. In each case, the rationale of CSF diversion was to avoid persistent CSF leak post-operatively.

### 2.2. CSF diversion regime

The primary role of the automated CSF diversion device in our protocol was to precisely modulate the fluid pressures across the operative site for a period of 5–7 days, complementing the surgical repair in situ. Patient-specific CSF diversion regimes were primarily informed by the calculation of individual CSF production rates.

#### 2.2.1. Equipment set-up

**Figure 1.**
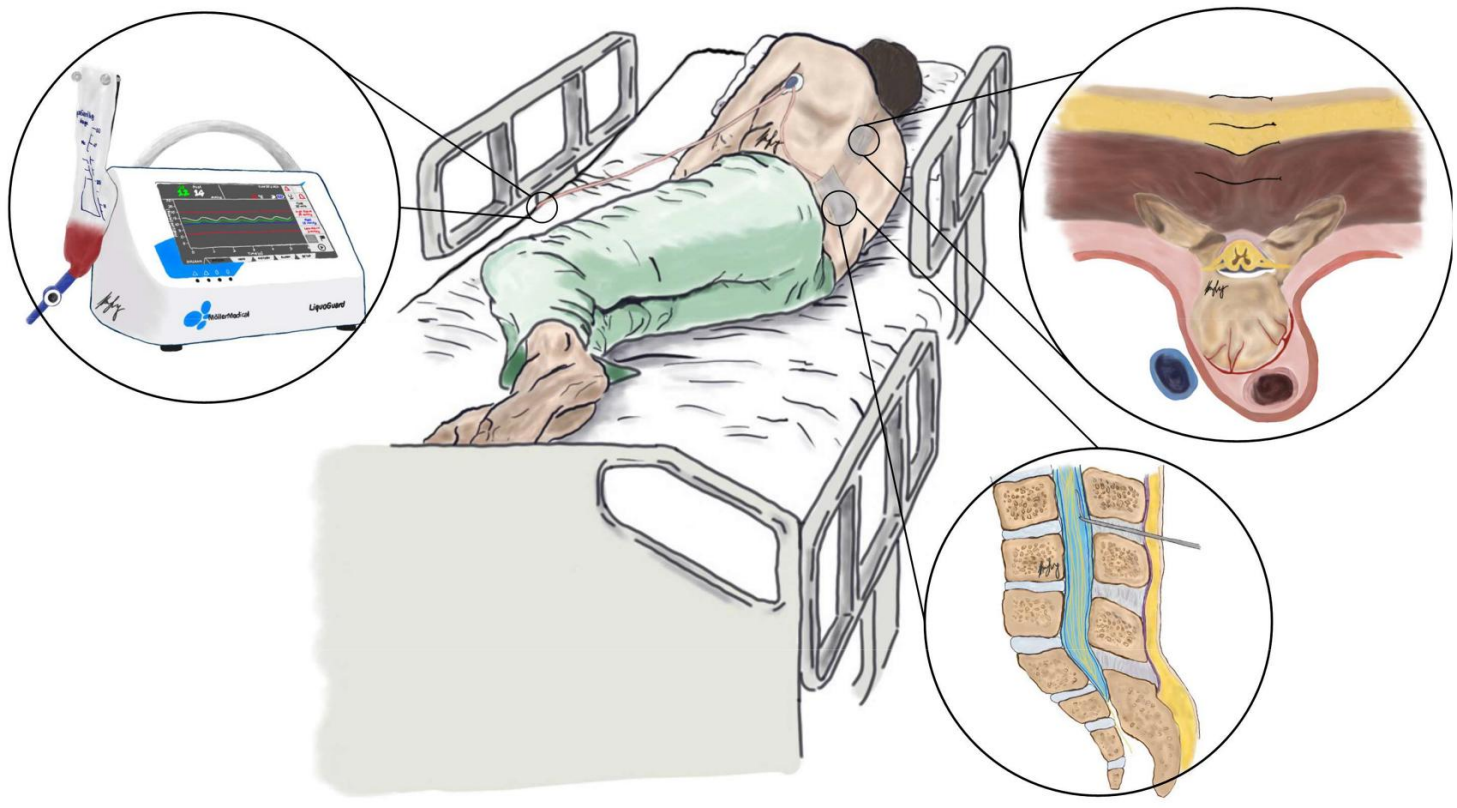
Illustration of the instituted wound repair & CSF diversion protocol. A mid-thoracic wound, closed in layers is demonstrated. Inferiorly, a lumbar drain is in-situ, and is connected to LiquoGuard7® tubing which has an in-line sensor placed at the axilla (approximating the level of the wound in the axial plane). Distal to the sensor, the tubing connects to a portable LiquoGuard7®, by the patient’s bedside.

A LiquoGuard7® system (Möller Medical, Fulda, Germany) is connected to the lumbar drain (Medtronic® Duet epidural catheter, USA). The device uses a peristaltic tube pump to drain CSF continuously, controlled by a computer with modifiable parameters for CSF pressure (pressure drainage threshold or Pset; hourly drainage volume or Vset; and pressure-based alarm settings). The computer receives pressure inputs via a fluid transducer, using a portable external reference sensor levelled to the area of interest. In our protocol, the portable sensor was affixed to the patient using an electrocardiogram sticker at the level of the operative wound, in line with the external auditory meatus (Figure 1).

#### 2.2.2. CSF production rate calculation

The calculation of CSF production rate (prCSF) is based on the work of Ekstedt et al in 1977 and 1978 who established that in a completely supine individual, in the absence of obstruction, the CSF pressure is homogenously uniform throughout the fluid compartments of the brain and spinal cord.^28–30^ Under such physiological conditions, when CSF pressure is externally controlled to a value below venous pressure, no CSF is absorbed in the venous circulation and thus CSF can be collected at its formation rate through an external drain.^28–32^ Electron microscopic studies, immunohistochemical studies and confocal microscopic studies of the arachnoid villi, nasal mucosal venous plexus and fluid pores of the pial membranes demonstrated a one-way, pressure-dependent bulk flow of CSF through the fluid pores. CSF absorption into the venous compartment, therefore, only occurs when CSF pressures exceeds venous pressure.^33^ Ekstedt in 1977 and 1978 used infusion of artificial CSF constantly for half hour in normal humans at pressure below intracranial venous pressure and hence prevented CSF absorption by the intracranial venous compartment. Using a lumbar drain, he was therefore able to collect CSF at its ‘normal’ baseline production rate, and believed to be between 16–34ml/hour. The LiquoGuard7®’s ability to maintain CSF pressure at a pre-set value facilitates the calculation of the prCSF based on the same principle. In our protocol, the Pset was set at 0mmHg (below venous pressure) and Vset was raised to 150ml/hour (maximum available setting). After allowing 20 minutes for CSF flow rates to stabilise, the volume of drainage over additional 10 minutes was used to calculate hourly prCSF using the computer software of the LiquoGuard7®.^32^ This was performed for each case, for three consecutive days (at the same time each day), to generate a mean prCSF value.

### 2.3. Data collection

CSF production metrics were collected prospectively, whilst the rest of the data was collected retrospectively. This included age, sex, relevant past medical history, spinal pathology, imaging, operative details and post-operative outcomes.

### 2.4. Data analysis

Pre-processing included re-categorizing free text into common data categories. Descriptive statistics were calculated (Microsoft Excel, Version 16.54) to summarize baseline characteristics (demographic, pathology, and operative characteristics), prCSF (mean, standard deviation) and surgical outcomes.

## 3. Results

### 3.1. General characteristics

In total, 3 patients were included in the series. A summary of demographics, medical background and operative details is provided below and in Table 1. Common to all of these cases are large dural defects, with a high flow intra-operative CSF leak in the context of a variety of pathologies (degenerative, benign tumour, malignant tumour), requiring multilayer reconstruction and CSF diversion.

**Table 1.** Summary of the relevant background, operative and outcome details for each case included in this illustrative series.

Case summary	Demographic	Relevant past medical history	Pathology	Operation(s) performed	Operative closure	CSF diversion protocol	Days of CSF diversion	Post-operative outcome
Degenerative Thoracic Disease	53-year-old, female	Nil	Giant calcified T7/T8 disc prolapse	Approached via a right-sided thoracotomy. Followed by resection of the right T7-T8 pedicle-rib complex, T7/T8 discectomy, and T7 & T8 partial corpectomy. A durotomy was performed to allow en-bloc resection of the prolapsed disc found to be traversing the dura.	Dural repair using Duragen and Evicel. Wound closure in layers, with a chest drain left in situ. A lumbar drain was placed after the procedure, under the same GA.	Initially, a pressure-led regime of 0cm H2O via a Becker drainage system. A LiquoGuard was then attached with settings of Pset: −1, Vset 10. Subsequent regimes involved a progressive increase in Vset parameters (10, 20, 30, 40, 50) to match CSF production with a final Vset of 50. CSF production rate: mean 140ml/hr (SD 5)	5 days (then the lumbar drain blocked). Bed rest, with the head of bed greater than 45 degrees was advised.	No wound, CSF leak or neurological complications. Mobilised 75 hours post-op.
Benign Thoracic Spine Tumour	44-year-old, female	Ex-smoker	Recurrent thoracic meningioma (WHO Grade 1)	Approached via a right T8-T9 costotransversectomy. Followed by a right T7-T9 hemilaminectomy, gross total resection of the tumour with resection of the posterior and anterior dura (Simpson Grade 0). Fusion was achieved via a right sided screw fixation at T6-T10.	Dural repair using Durapair, Haemopatch and Evicel. Wound closure in layers. A lumbar drain was placed after the procedure, under the same GA.	A LiquoGuard was attached post-operatively with an initial regime of Pset 3 and Vset 50. The Pset was progressively reduced to −5, and then −14, in response to bilateral pleural effusions (likely CSF). CSF production rate: >150ml/hr	7 days. Bed rest, with the head of bed greater than 45 degrees was advised.	Resolution of pleural effusions. No wound, CSF leak (supported by radiological evidence) or neurological complications. Mobilised 37 hours post-op.
Malignant Thoracic Spine Tumour	54-year-old, female	Numerous cycles of radiotherapy (maximum dose) & chemotherapy for metastatic uterine leiomyosarcoma Inferior vena cava thrombus (on Apixaban)	Paravertebral metastasis extending from the thoracic cavity into the spinal canal at T4-T5.	First operation: Midline approach, with bilateral pedicle screw insertion at T3 & T6. Followed by a T4-T5 laminectomy, left T5 pediculectomy, and debulking of the intraspinal tumour. No dural repair. Wound closure in layers. Second operation: Approached via the previous incision, inspection for CSF leak (in which a discrete leaking point was not identified) but diffuse CSF egress was found.	For the second operation, dural repair was performed using Duragen, Tisseel, Spongostan. Wound closure in layers. A lumbar drain was placed before the procedure, under the same GA.	A LiquoGuard was attached intra-operartively with an initial regime of Vset 2 and Pset −5 (to keep the drain patent). Post-operatively, a pressure-led regime of Pset 0 and Vset 150 was used. The Pset was reduced to −2 due to bypassing of CSF around the lumbar drain. CSF production rate: >150ml/hr	7 days. Head of bed greater than 45 degrees was advised.	No wound leak or CSF leak (supported by radiological evidence), and no neurological complications. Mobilised 35 hours post-op.

CSF: cerebrospinal fluid, GA: general anaesthetic, PSET: CSF pressure drainage setting (mmHg), VSET: CSF volume drainage setting (mls/hr).

### 3.2. Case series

#### 3.2.1. Case 1 – degenerative thoracic spine disease

**Figure 2.**
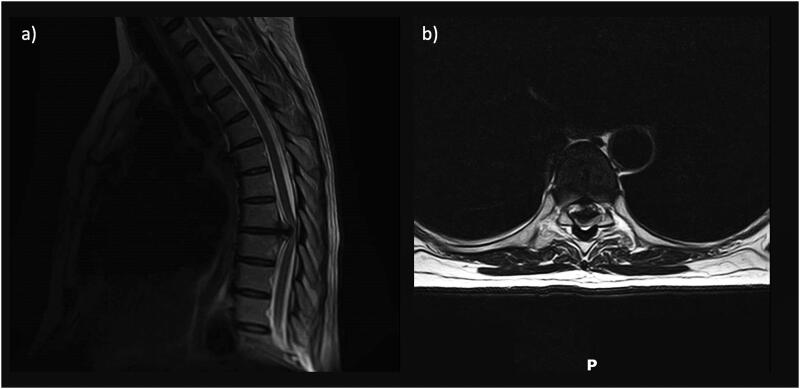
Case of degenerative thoracic disease, with pre-operative MRI scans displaying the large calcified T7-T8 disc prolapse in sagittal (a) and axial (b) profiles.

A 53-year-old female was admitted with evidence of thoracic myelopathy secondary to a large calcified T7/T8 disc prolapse (Figure 2). She underwent a decompression via right-sided thoracotomy, right T7-T8 pedicle-rib complex resection, T7/T8 discectomy and partial corpectomy. The disc was found to have traversed the dura, and therefore durotomy was required for full resection. This dural defect was repaired via dural replacement (Duragen®) with tissue glue (Evicel®), and superficial wound closure was performed in layers in a standard fashion. A chest drain was left in situ, and a lumbar drain was placed under the same general anaesthetic in response to the CSF leak with an aim to decrease CSF pressure differential across the dural repair and prevention of post-operative CSF leakage/CSF fistula formation.

The lumbar drain was initially attached to a Becker drainage system with a pressure-led regime (at 0cm H2O, levelled at the level of the thoracic wound). A LiquoGuard7® was available on day 1 post-op, and was again set to a pressure-led regime (pressure set at −1 mmHg, with a volume drainage setting of up to 10mls/hr). The mean prCSF was 140ml/hour, ± 5 SD. Resultantly, the maximum volume drainage setting was increased in 10ml/hr increments daily, up to a cautious setting of 50mls/hour. CSF drainage was maintained for 5 days until the lumbar drain blocked. Bed rest was implemented, with the head of the bed kept at 45 degrees. The volume of CSF drained per 24 hours was 500ml on day one, 750ml on day two, 1000ml on day three and 1200 ml each day on day four and five. The patient had no wound-related issues, post-operative CSF leak or pseudomeningocele.

#### 3.2.2. Case 2 – benign thoracic spine tumour

**Figure 3.**
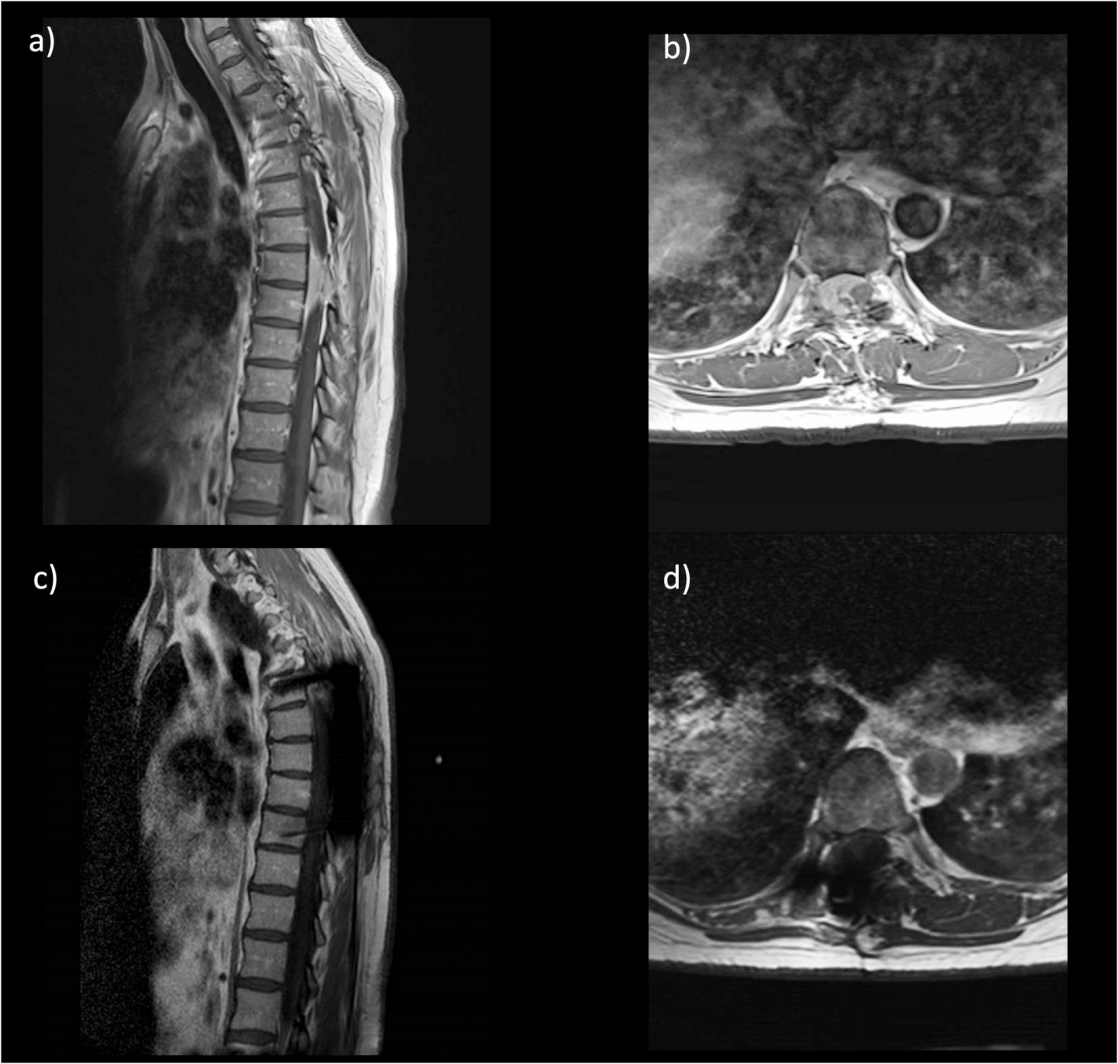
Case of benign thoracic spine tumour with pre-operative contrast MRI images showing the recurrent thoracic meningioma in sagittal (a) and axial (b) views. Post-operative images show satisfactory resection in sagittal (c) and axial (d) views.

**Figure 4.**
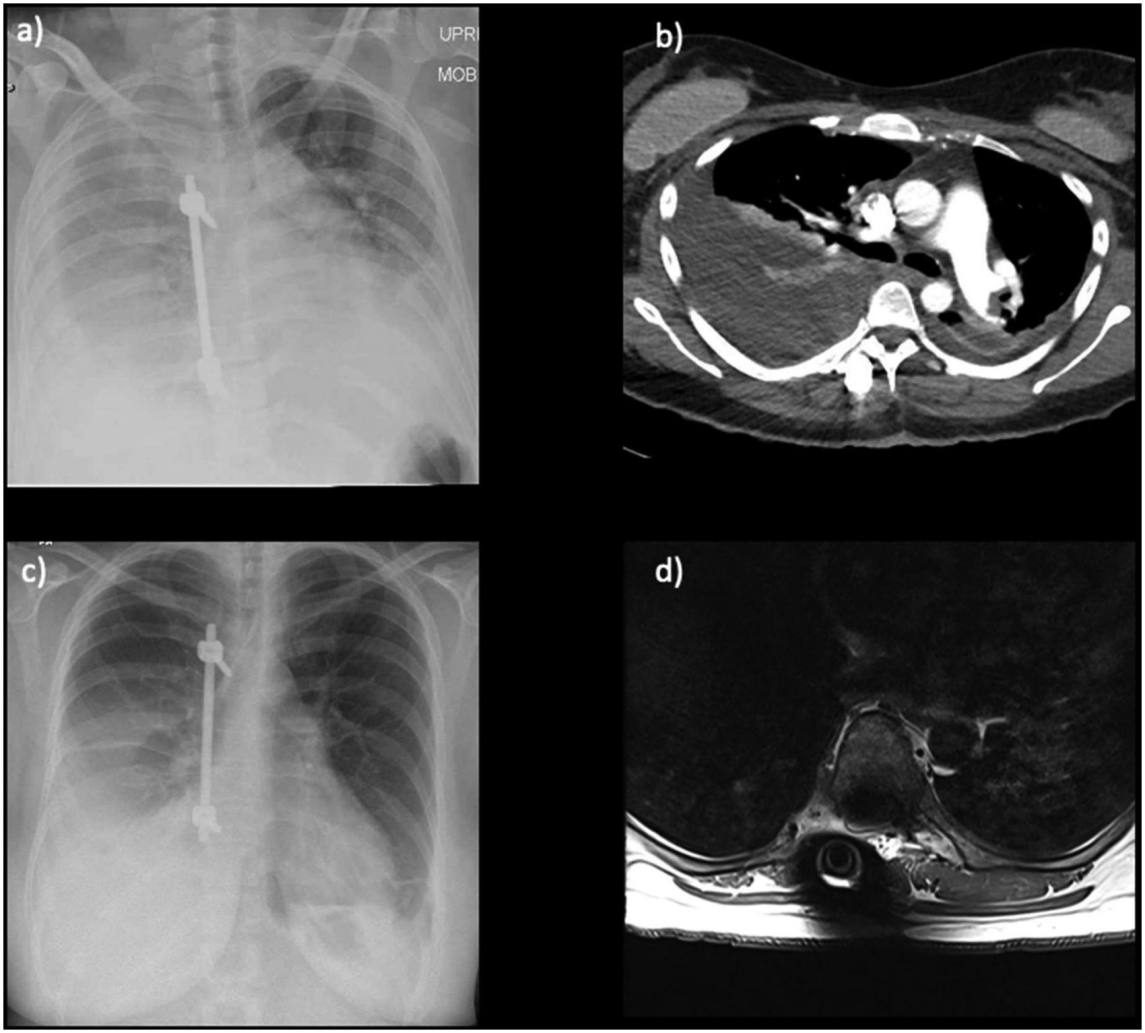
Case of benign thoracic spine tumour with immediate post-operative chest x-rays (a) and CTPA (b) showing pleural effusion. After a period of Liquoguard-driven lumbar drainage, a repeat chest x-ray 5 days later (c) shows interval improvement, and a delayed MR 4 months later shows resolution of effusion at the lung bases (d).

A 44-year-old female was admitted with thoracic myelopathy secondary to a recurrent T7-T9 meningioma (Figure 3). She had a gross total resection of this WHO Grade 1 tumour via right T8-T9 costotrasversectomy and right T7-T9 hemilaminectomy (to allow access to the anterolateral aspect of the tumour, which was adherent to the cord), with fusion T6-T10 on the right side. As the tumour was adherent to the dura, the anterior and posterior dura was circumferentially resected along with the tumour to achieve a Simpson Grade 0 resection and optimise the oncological outcomes in this young patient. This expectedly resulted in a high-flow CSF leak, fistulating with the pleural cavity. The dura was repaired with Durapair®, Haemopatch® and Evicel®, and superficial wound closure was performed in layers in a standard fashion. A lumbar drain was placed under the same general anaesthetic in response to the CSF leak, and was connected to a LiquoGuard7®. prCSF rate was consistently found to be greater than 150ml/hour (i.e., greater than the maximum measurable value) on 3 consecutive days. The starting CSF drainage protocol was a pressure-led regime (pressure set at 3 mmHg, with a cautious volume drainage setting of up to 50mls/hr). The known CSF-pleural fistula subsequently resulted in a significant right-sided pleural effusion on imaging (Figure 4). The commonly employed approach for this complication is to proceed with the insertion of a chest drain, which can perpetuate the CSF leak into the thoracic cavity by increasing intra-thoracic negative pressure, and lead to poorly controlled high-volume CSF drainage (with life-threatening risks such as tonsillar descent into the foramen magnum and posterior fossa haemorrhage). Therefore, alternative management via tailored CSF diversion was used. The pressure set parameter was progressively decreased to −5 mmHg and then −14mmHg to overcome the negative intra-thoracic pressure, with changes guided by serial imaging. CSF was diverted in this fashion for a total of 7 days before the lumbar drain was removed. The volume of CSF drained per 24 hours was 1200 ml each day for 7 days. This regime achieved complete resolution of effusion (Figure 4), allowing early mobilisation without significant respiratory compromise, any further operations, wound-related issues, post-operative CSF leak or pseudomeningocele.

#### 3.2.3. Case 3 – malignant thoracic spine tumour

**Figure 5.**
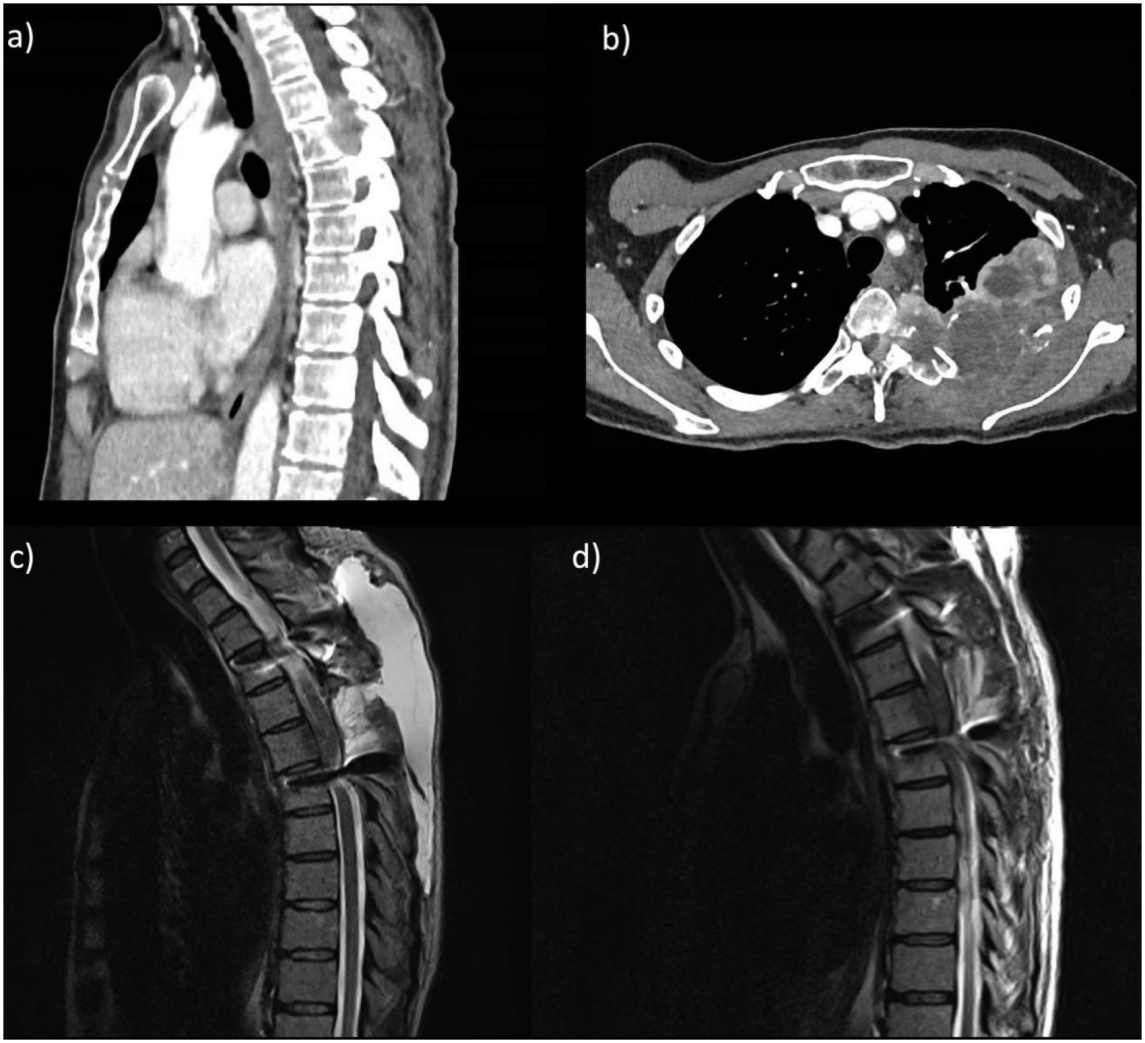
Case of malignant thoracic spine tumour with pre-operative CT images showing the paravertebral metastatic uterine leiomyosarcoma in sagittal (a) and axial (b) views. An MRI sagittal view of the CSF leak after the first operation is displayed in c), with a subsequent MRI after operative exploration, repair and CSF diversion shown in d).^28^^,^^29^

A 54-year-old female with left T4-5 paravertebral metastasis causing spinal cord compression, refractory to a maximum dose of radiotherapy (Figure 5). She underwent bilateral T3 & T6 pedicle screw placement, with T4-T5 laminectomy, T5 unilateral pediculectomy and intraspinal tumour debulking. No overt intra-operative CSF leak was noted, although the tumour infiltration of the paraspinal structures was extensive, including anterior to the spinal cord (on the margin of but beyond the resection cavity). In the primary operation, closure was performed in layers without dural reconstruction. Two weeks later, the patient represented with radiological evidence of a CSF leak, causing a large pseudomeningocele (Figure 5). This required operative exploration (no leaking point or medial screw breach identified, although diffuse and active CSF egress was noted), repair (with Duragen®, Tisseel® and Spongostan®), with lumbar drain insertion before this under the same GA. This drain was immediately attached to a LiquoGuard7® system, with intra-operative parameters set to maintain drain patency (volume drainage of up to 2mls/hr, to maintain a pressure of −5 mmHg at the level of the wound). Again, the prCSF was consistently found to be greater than 150ml/hour (i.e., greater than the maximum measurable value) on 3 consecutive days. Based on these findings the LiquoGuard7 settings were changed to a pressure-led regime set at 0 mmHg, levelled at the level of the thoracic wound, with maximum drainage of up to 150mls/hr. During this time, the head of the bed was kept at 45 degrees at least. The patient was able to mobilise earlier, remained neurologically well and experience neither low-pressure symptoms nor displayed any signs of over drainage. The pressure setting was subsequently lowered to −2 mmHg due to bypassing of CSF around the lumbar drain. The lumbar drain malfunctioned after 7 days and was removed. The volume of CSF drained per 24 hours was 3600 ml each day for 7 days. Post-operatively, she continued her oncology treatment, without any further wound issues or CSF leak.

## 4. Discussion

### 4.1. Principal Findings

Through an illustrative case series of three distinct spinal pathologies with high-flow intra-operative CSF leak, we have demonstrated the utility of patient-specific CSF diversion, based on individual CSF flow rates, to assist with layered operative closure. Furthermore, we propose automated CSF pressure and volume control via devices such as the LiquoGuard7® for more precise control of pressures across dura defects and operative wounds. Our experience in this regard was iterative, and central to all regimes used was pressure-led programming with sensors calibrated to the level of wound. Drainage regimes were tailored for CSF production rates, reduction of extra-axial CSF collections (e.g. pleural effusions) and drain catheter malfunction – displaying the versatile utilities of automated CSF drainage systems. Although maximally allowed drainage volumes were initially 50ml/hr, we allowed drainage up to 150ml/hr in selected and carefully monitored scenarios, which did not result in significant over-drainage sequelae. We hypothesize this is due to the recorded CSF hyperproduction in all 3 of these cases (Case 1: mean 140ml/hr, Case 2: mean >150ml/hr, Case 3: mean >150ml/hr). Emerging research suggests CSF production rates may increase as a physiological response to the dural breach and rebound intracranial hypertension may occur after the repair of CSF leaks.^22^^,^^23^ These factors support the use of CSF diversion in theory in the context of spinal wound closure at high risk for post-operative CSF leak, which is reflected in contemporary, albeit heterogeneous, spinal surgery practice.^1^^,^^3^^,^^9^^,^^10^^,^^15^^,^^16^ In our experience, selected cases stand to benefit from this intersection of patient-specific data and novel smart technology, allowing tight pressure control at the operative site and ultimately satisfactory surgical outcomes. In our developing practice, these cases will include those at high risk of a post-operative leak, for example, planned large dural defects requiring reconstruction (e.g. large meningioma resection) or cases at high risk of inadvertent complex dural defects (e.g. previous multiple revisions).

Poiseuille’s law has been used to describe the flow of CSF across an iatrogenic dural defect.^34^^,^^35^ Here, reducing the radius of the defect dramatically reduces flow (e.g. via primary dural repair, or reconstruction using alternative materials). Resistance across the defect can also be increased through the layering of reconstructive materials and external pressure on wounds (e.g. pressure dressings). The pressure gradient across the defect (intradural to extradural) is reduced through patient positioning (upright at C-spine, 45 degrees at T-spine, lying at L-spine) and tailored CSF diversion according to CSF flow rate (e.g. subfascial or subarachnoid lumbar drain).^34–36^ These methods likely act synergistically, with pressure modulation via positioning and drainage representing practical modifiable factors in the post-operative care of these patients.^34^^,^^35^ Furthermore, histological studies suggest the presence of CSF in a wound cavity can inhibit normal wound-healing processes.^37^^,^^38^ Using our combined patient-specific CSF diversion and layered wound closure regime, no cases in this consecutive series required further intervention for CSF fistulae repair (including for pleural CSF effusion), wound breakdown or infection, whilst reaping the benefits of early post-operative mobilisation.

### 4.2. Findings in the context of the literature

Automated CSF pressure and drainage control using LiquoGuard® technology have been used for various neurosurgical indications, from managing complex hydrocephalus and traumatic brain injury to preventing spinal cord ischaemia. There are numerous benefits of LiquoGuard® over conventional drainage systems (i.e., closed passive drainage systems such as the Becker drain). These include allowing early patient mobilization (as the system is levelled via a portable sensor fixed to the patient and therefore moves with the patient, rather than a fixed level).^25^ This feature also prevents the complication of over-drainage or under-drainage, frequently encountered with passive drainage systems. Additionally, mechanical complications may be reduced through using an automated drainage system, including reduced drain blockage and CSF leak at the drain site – both likely due to the continuous drainage implemented by these systems, rather than intermittent drainage seen with manual drainage systems.^39^

Furthermore, precise measurement and control of CSF pressure result in fewer pressure peaks and granular pressure and drainage data which can be integrated into management regimes.^24^^,^^26^^,^^40^^,^^41^ This CSF pressure regulation has been used in tandem with tight blood pressure control, to modulate spinal cord perfusion and thus prevent cord ischaemia during endovascular aortic repair.^40–43^ The system also theoretically reduces staff workload through automatic detection of drain dysfunction (e.g. occlusion, disconnection, pressure discrepancies) and elimination of the need for manual intermittent drainage (and recording of this drainage).^26^^,^^39^^,^^40^ However, these systems are expensive, not widely available, require staff training, and are not yet supported by high-level comparative evidence.

### 4.3. Strengths and Limitations

The strengths of this study lie in the novelty of patient-specific automated CSF drainage, and the variety of use cases within spinal surgery displayed. Limitations of this illustrative study include small sample and non-comparative design. The current prCSF protocol and LiquoGuard7® allow for a maximum volume drainage of 150ml/hour, and therefore flow rates above 150ml/hour cannot be calculated. Similarly, measurements are taken for 10 minutes for practical reasons but are extrapolated to hourly rates, which in theory could under or overestimate the true hourly rate. Future studies will require larger, prospective, controlled studies to refine CSF drainage regimes and assess the comparative benefit of the LiquoGuard7® system.

## 5. Conclusions

Automated patient-specific cerebrospinal fluid drainage may have a role in the closure of complex spinal wounds with large dural defects and high-flow intra-operative CSF leaks. These systems may reduce post-operative CSF leakage from the wound or into adjacent body cavities. Further larger studies are needed to explore the comparative benefits and cost-effectiveness of these devices in contemporary spinal neurosurgery.

## Ethics and informed consent

Institutional ethics was attained for the study and informed consent was taken from all patients included.

## Details of previous presentations

Previous poster presentation at the Surgical Research Society Meeting (Nottingham, UK) on 02.23.23

## Data Availability

Data available upon reasonable request
